# Pharmacokinetics of anti-infective agents during CytoSorb hemoadsorption

**DOI:** 10.1038/s41598-021-89965-z

**Published:** 2021-05-18

**Authors:** Antoine G. Schneider, Pascal André, Joerg Scheier, Monika Schmidt, Heiko Ziervogel, Thierry Buclin, Detlef Kindgen-Milles

**Affiliations:** 1 Adult Intensive Care Unit, BH08-610, Centre Hospitalier Universitaire Vaudois (CHUV), 46, Avenue du Bugnon 1011, Lausanne, Switzerland; 2 Faculty of Biology and Medicine, University of Lausanne, Lausanne, Switzerland; 3 Clinical Pharmacology, Centre Hospitalier Universitaire Vaudois (CHUV), Lausanne, Switzerland; 4 CytoSorbents Europe GmbH, Berlin, Germany; 5 Medical Competence Center Berlin/Brandenburg C/o HCx Consulting GmbH, Wendisch Rietz, Germany; 6 Department of Anesthesiology, University Hospital Düsseldorf, Düsseldorf, Germany

**Keywords:** Experimental models of disease, Preclinical research, Renal replacement therapy

## Abstract

Cytokine hemoadsorption might be beneficial in patients with sepsis. However, its effect on anti-infective agents' disposition remains largely unknown. We sought to determine the influence of hemoadsorption on the pharmacokinetics of common anti-infective agents. This is an interventional experimental study, conducted in 24 healthy pigs. Animals were randomly allocated to either hemoadsorption (cases) or sham extracorporeal circuit (controls) and to drug combinations (3 cases and 3 controls for each combination). Hemoadsorption was performed with CytoSorb (CytoSorbents Corporation, USA). We evaluated 17 drugs (clindamycin, fluconazole, linezolid, meropenem, piperacillin, anidulafungin, ganciclovir, clarithromycin, posaconazole, teicoplanin, tobramycin, ceftriaxone, ciprofloxacin, metronidazole, liposomal amphotericin B, flucloxacillin and cefepime). Repeated blood sampling from the extracorporeal circulation (adsorber inlet/outlet, sham circuit) was performed over six hours following administration. Total clearance and adsorber-specific clearance were computed. Hemoadsorption was associated with increased clearance of all study drugs, except ganciclovir. Its impact on total body clearance was considered as moderate for fluconazole (282%) and linezolid (115%), mild for liposomal amphotericin B (75%), posaconazole (32%) and teicoplanine (31%) and negligible for all other drugs. Hemoadsorber clearance declined over time, with even delayed desorption for beta-lactams. It was moderately correlated with drug's lipophilicity (p = 0.01; r^2^ = 0.43). Hemoadsorption with CytoSorb appears to increase to a clinically significant extent the clearance of five among 17 tested anti-infectives. Studies in human patients are required to confirm the need for dosage adjustment of these agents.

## Introduction

Sepsis is a major health issue worldwide, affecting close to 50 million individuals each year and leading to 11 million deaths^1^. Recognition of sepsis was identified as a global health priority by the World Health Organization in 2017. Sepsis results from a dysregulated host response to infection^2^. Activation of both complement and coagulation systems lead to the massive release of pro- and anti-inflammatory cytokines in the blood, a phenomenon sometimes referred to as "cytokine storm"^3^. This response through systemic hypotension, microcirculation alterations, endothelial lesions, as well as metabolism modulation, ultimately leads to cellular apoptosis, organ failure and death.

CytoSorb (CytoSorbents Corporation, NJ, USA), a hemoadsorption device, has been marketed and licensed for extracorporeal cytokine removal within the European Union since 2011^4^. CytoSorb cartridges can easily be inserted in extra-corporeal circulation circuits. They contain biocompatible polystyrene divinylbenzene copolymer beads coated with polyvinylpyrrolidone capable of removing molecules of mid-molecular weight using a combination of hydrophobic or ionic interactions as well as hydrogen bonding^5,6^.

The level of evidence supporting the use of CytoSorb in septic shock remains low and largely observational^7–11^. In their latest statement, experts from the Surviving Sepsis Campaign urged for further research and did not recommend for or against blood purification techniques^12^. In this context, safety parameters are of particular importance for the decision to initiate such therapy in a patient. So far, post marketing surveillance and data from published literature has not suggested major adverse events apart from occasional thrombocytopenia. However, the potential removal of life-saving medications such as antibiotics in sepsis requires particular attention^4^. Indeed, little is known about the effect of CytoSorb on anti-infectives' pharmacokinetics. In vitro models have confirmed its ability to remove some anti-infectives from the blood^13,14^. However, these one-compartment models have numerous limitations, and their results might not be translatable to humans. Authors have reported cases suggesting a minor influence on serum blood levels^15–17^. However, these reports lack consistency and reproducibility.

Accordingly, we have designed an experimental animal study to determine the influence of hemoadsorption with CytoSorb on the pharmacokinetics of anti-infective agents commonly prescribed in sepsis.

## Materials and methods

### Ethics and legal aspects

The experimental study was conducted in a medical competence center located in Wendisch Rietz (Germany), in compliance with German law for animal protection.

### Animal preparation and monitoring

We included 24 healthy German landrace pigs (10 female, 14 male) with a body weight of 45–60 kg (mean 52.3 kg). The pigs were pre-medicated with an initial intramuscular injection of ketamine (15 mg/kg), midazolam (0.25 mg/kg) and azaperon (6 mg/kg). Sedation was maintained using a continuous intravenous infusion of ketamine (10 mg/kg/h) and midazolam (0.5 mg/kg/h). Pancuronium was administered for muscle relaxation as required. Anticoagulation with heparin was administered. Surgical tracheostomy was performed to allow conventional mechanical ventilation (Evita XL or Oxylog 3000; Draegerwerk AG & Co KG Luebeck, Germany). Surgical catheterization of the carotid artery and jugular vein was performed (respectively Avanti and Avanti plus sheath introducer; Cordis Miami, FL, USA) to allow for blood pressure monitoring and enable fluid and medication administration respectively. Vital signs were monitored throughout the experiment (Philips M3046A Philips IntelliVue MP5; Philips, Amsterdam, the Netherlands). Finally, a 12 Fr double lumen catheter (Dualyse expert; Vygon GmbH & Co KG, Aachen, Germany) was inserted into either the femoral or jugular vein to enable extracorporeal circulation. All animals were euthanized at the end of the experiment while fully sedated, by simultaneous intravenous potassium chloride and pancuronium administration.

### Experimental design

Four experiments were conducted corresponding to four anti-infectives combinations. These combinations as well as drug dosing and injection patterns are presented in Table 1. We tested beta-lactams (meropenem, piperacillin, ceftriaxone, flucloxacillin and cefepime), antifungals (fluconazole, anidulafungin, posaconazole and liposomal amphotericin B), aminoglycosides (tobramycin) and other type of anti-infectives (linezolid, clindamycin, ganciclovir, clarithromycin, teicoplanin, ciprofloxacin and metronidazole).

**Table 1:** Experimental groups.

Experiment	Drugs	Dosing	Intervention
1	Meropenem	2000 mg over 30 min	Sham (3 pigs) CytoSorb (3pigs)
	Linezolid	600 mg over 30 min	
	Fluconazole	800 mg over 40 min	
	Clindamycin	1200 mg over 40 min	
2	Piperacillin	6000/750 mg over 30 min	Sham (3 pigs) CytoSorb (3 pigs)
	Anidulafungin	200 mg over 60 min	
	Ganciclovir	400 mg over 60 min	
3	Ceftriaxone	4000 mg over 30 min	CytoSorb (3 pigs) Sham (3 pigs)
	Clarithromycin	500 mg over 60 min	
	Posaconazole	300 mg over 30 min	
	Teicoplanin	800 mg over 30 min	
	Tobramycin	320 mg over 30 min	
4	Ciprofloxacin	400 mg over 60 min	Sham (3 pigs) CytoSorb (3pigs)
	Cefepime	2000 mg over 30 min	
	Metronidazole	2000 mg over 30 min	
	Amphotericin B (liposomal)	150 mg over 30 min	
	Flucloxacillin	4000 mg over 30 min	

Pigs received different combinations of drugs, followed by initiation of an extra-corporeal circulation with (cases) or without (sham, control) a CytoSorb cartridge. Three animals were randomly allocated to each group. All drugs were administered intravenously one hour before extra-corporeal circulation initiation.

During each experiment, six animals were prepared as described above. After the administration of the anti-infective combination, animals were randomly allocated to either hemoadsorption with CytoSorb (cases) or sham hemoperfusion (control group) on a one to one ratio.

### Extracorporeal circulation

**Figure 1:**
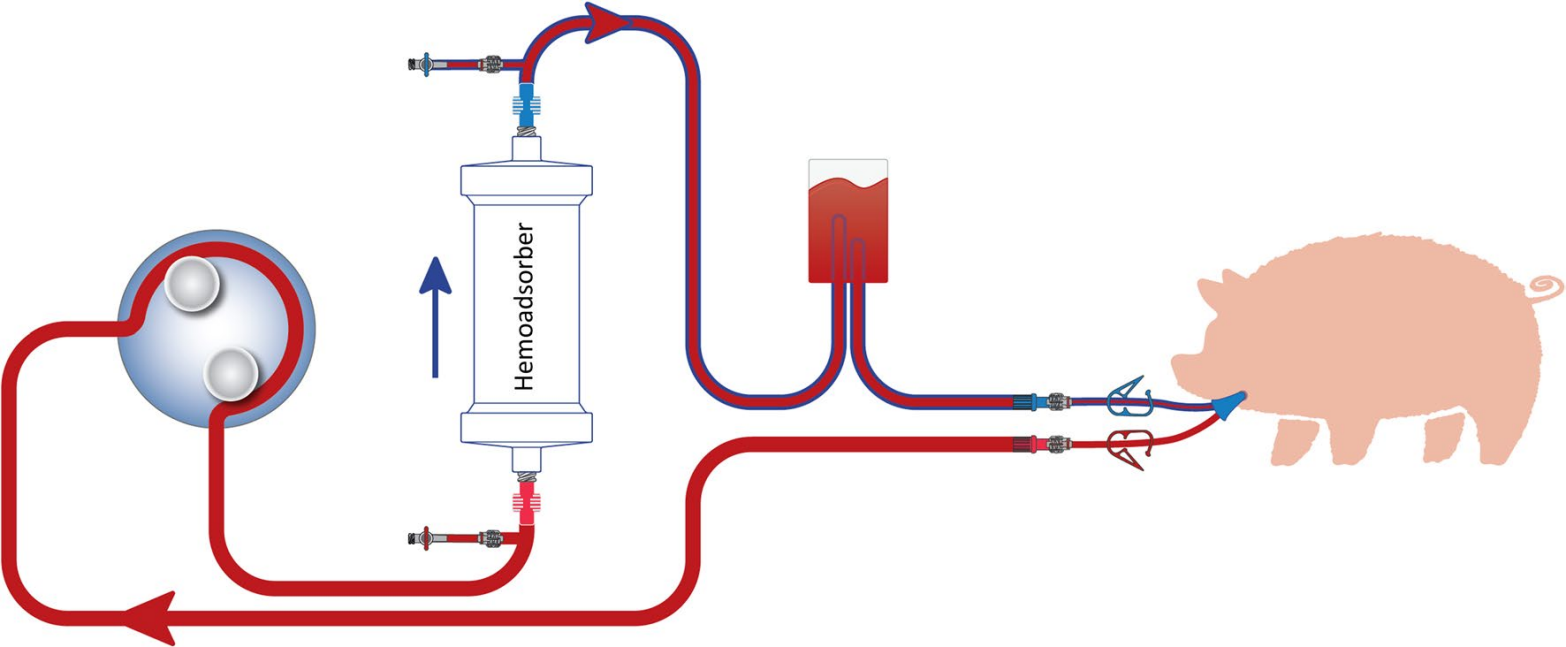
Extra-corporeal circuit. According to study protocol, 1 h following anti-infective administration, study animals were randomly allocated to either CytoSorb hemoadsorption or sham procedure on a 1:1 basis. For animals allocated to the sham procedure, a similar circuit without hemoadsorber was used.

A schematic representation of the extracorporeal circulation (ECC) is shown in Fig. 1. It was established in all animals with a dedicated device [BM11a, Baxter, Deerfield, IL, USA) and corresponding circuit (BM11-Lines-BA-HP (tube system set BM11-hemoperfusion adult set; VE17; BLD-clamp/VE); Baxter, Deerfield, IL, USA]. For animals allocated to the intervention group (Table 1), a CytoSorb cartridge was inserted in the ECC. For those allocated to the control group no cartridge was integrated (sham hemoperfusion). ECC was started one hour after the start of study anti-infective administration. Blood flow rate was kept between 150 and 200 mL/min throughout the experiment.

### Blood samples and laboratory analysis

Blood samples were collected before the initiation of the anti-infective infusion, then respectively 5, 30, 90, 250 and 330 minutes after adsorber/sham procedure initiation. For animals allocated to the intervention group, we collected two samples per time point: before (inlet) and after (outlet) the adsorber. For those allocated to the control group, we collected a single systemic sample (Fig. 1). All samples (approx. 9 mL) were drawn into EDTA (ethylenediaminetetraacetic acid) monovettes (Sarstedt AG & Co KG, Nuembrecht, Germany). They were then centrifuged at 30,000 rpm for 10 min, split into two 2 mL Eppendorf tubes (Eppendorf AG, Hamburg, Germany) and frozen at -20°C. Drugs plasma levels were determined quantitatively by liquid chromatography tandem mass spectrometry (LC-MS/MS) (MS/MS: API 2000 or API 4000; Sciex, Nieuwerkerk aan den Ijssel, The Netherlands; HPLC pump: Agilent Technologies, Santa Clara, CA, USA; CTC autosampler: Sciex) in a human medical laboratory (Bioscientia GmbH, Ingelheim, Germany).

### Pharmacokinetic parameters calculations

Pharmacokinetics parameters were computed individually for each study drug and each animal using standard non-compartmental calculations and considering first order elimination kinetics. All calculations were performed using Microsoft Excel 2016 (Microsoft Corp., Redmond, WA, USA).

The areas under the plasma drug concentration curves (AUC) were calculated using the log-trapezoidal rule based on the last measurement (AUCo-last) and with extrapolation to infinity assuming a constant elimination rate (AUCo-inf). Total clearance (CL_tot_) could be deduced from the dose (D):

$${\text{CL}}_{{{\text{tot}}}} = {\text{D}}/{\text{AUCo-inf}}$$

Total amount eliminated during the study period (D_TE_) was calculated using the following formula:

$${\text{D}}_{{{\text{TE}}}} = {\text{D}} \times {\text{AUCo-last}}/{\text{AUCo-inf}}$$

In addition, for animals allocated to the adsorber arm, adsorber's instantaneous removal amount (D_IR_) were evaluated based on the following formula:

$${\text{D}}_{{{\text{IR}}}} = {\text{Q}}_{{\text{P}}} \times \left( {\left[ {{\text{inlet}}} \right] - \left[ {{\text{oulet}}} \right]} \right)$$

where Q_p_ is the effective plasmatic flow (hematocrit considered to be 40%), [inlet] the drug concentration in the pre-adsorber sample and [outlet] the drug concentration in the post adsorber sample. Cumulative removal attributable to the adsorber during study period (D_CC_) was calculated using the log trapezoidal rule on instantaneous removal rates. Finally, overall adsorber clearance during study period (CLc) could be estimated:

$${\text{CL}}_{{\text{C}}} = {\text{CL}}_{{{\text{tot}}}} \times {\text{D}}_{{{\text{CC}}}} /{\text{D}}_{{{\text{TE}}}}$$

For each drug, we computed the mean clearances with standard deviation by study group. Finally, we calculated the relative increase in overall animal total clearance brought by the hemoadsorber clearance (percent):

$${\text{Increase}}\;\left( \% \right) = {\text{CL}}_{{\text{C}}} /\left( {{\text{CL}}_{{{\text{tot}}}} - {\text{CL}}_{{\text{C}}} } \right) \times {1}00$$

### Clearance interpretation

We assessed the impact of the adsorber on drug clearance based on the classification proposed by the WHO Committee for Proprietary Medicinal Products (CPMP) for drug inhibitors and inducers according to potency^19^. Weak inducers increase clearance by >25%, moderate inducers by >100% and strong inducers by >400%. A mild effect was thus retained when the adsorber increased the baseline drug clearance by >25%, a moderate effect by >100% and a strong effect by >400%.

### Determinants of hemoadsorber clearance

To evaluate the impact of drugs’ physico-chemical properties on adsorber removal, we assessed their correlation with calculated adsorber clearance. For each study drug, we tested octanol–water partition coefficient (logP), octanol–water distribution coefficient (logD), acid dissociation constant at logarithmic scale (pKas), molecular electric charge (positive, negative, neutral or zwitterion), protein binding or molecular weight. These values were obtained using the Chemaxon online molecular library^20^.

### Statistical analyses

Between-groups comparisons were conducted using bilateral Student’s t-test. Correlation analyses were performed using the Pearson’s test. The strength of associations was assessed according to the correlation coefficient (ρ), considered to be strong (ρ > 0.8), fair (0.6 < ρ ≤ 0.8), moderate (0.4 < ρ ≤ 0.6) or weak (ρ ≤ 0.4). For all analyses, a p value < 0.05 was considered to be statistically significant. All analyses were performed using Graphpad Prism 8.3 (Graphpad Software Inc.).

### Ethics approval and consent to participate

The study was approved by the State Office for Occupational Safety, Consumer Protection and Health—Department of Consumer Protection (Brandenburg, Potsdam, Germany), approval number 2347-3-2018. According to the German animal protection law, no additional approval by an Ethics Committee was necessary. The present report was prepared following the ARRIVE guidelines for animal research^18^. 

## Results

Measured plasma concentrations and calculated clearances for all study drugs are reported in the supplemental material (Figs. S1–S4).

### Overall hemoadsorber clearance

**Figure 2:**
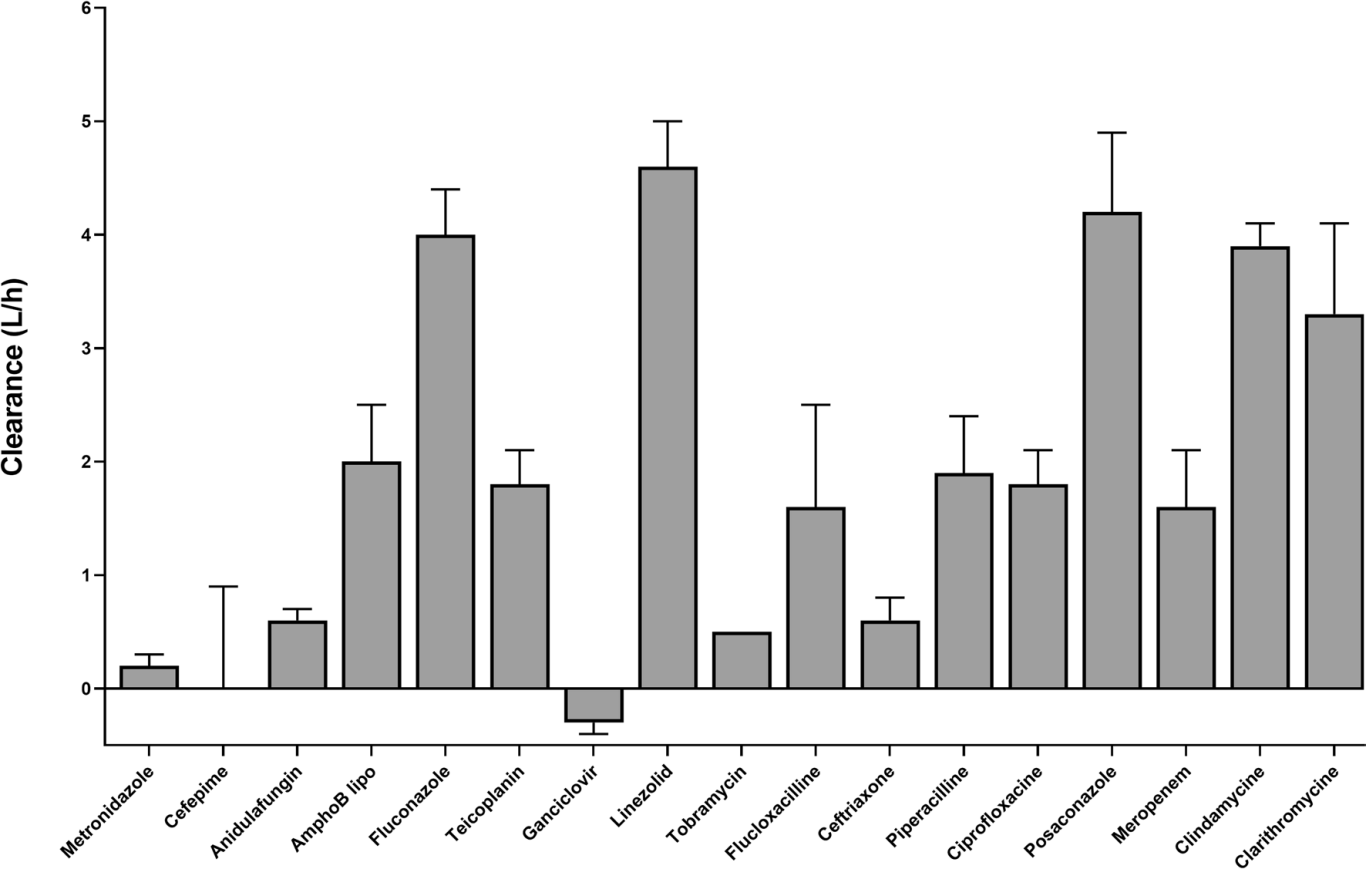
Total Adsorber Clearance. Clearance attributable to CytoSorb (mean and SD). Values were calculated based on pre and post adsorber measurements at different timepoints. AmphoB lipo: liposomal amphotericin B.

Fig. 2 depicts mean overall hemoadsorber clearance measured during the study period for each study drug under our experimental conditions. Mean clearance of almost all study drugs (except for ganciclovir) was positive indicating removal of the drug by the procedure. Mean clearance was > 3L/h for linezolid (4.6 L/h, SD 0.4), posaconazole (4.2 L/h SD 0.7), fluconazole (4.0 L/h SD 0.4), clindamycin (3.9 L/h SD 0.2) and clarithromycin (3.3 L/h SD 0.8). It was less than 2 L/h for all other molecules. A negative clearance (-0.3L/h) was observed for ganciclovir.

### Relative part of hemoadsorber over total body clearance

**Figure 3:**
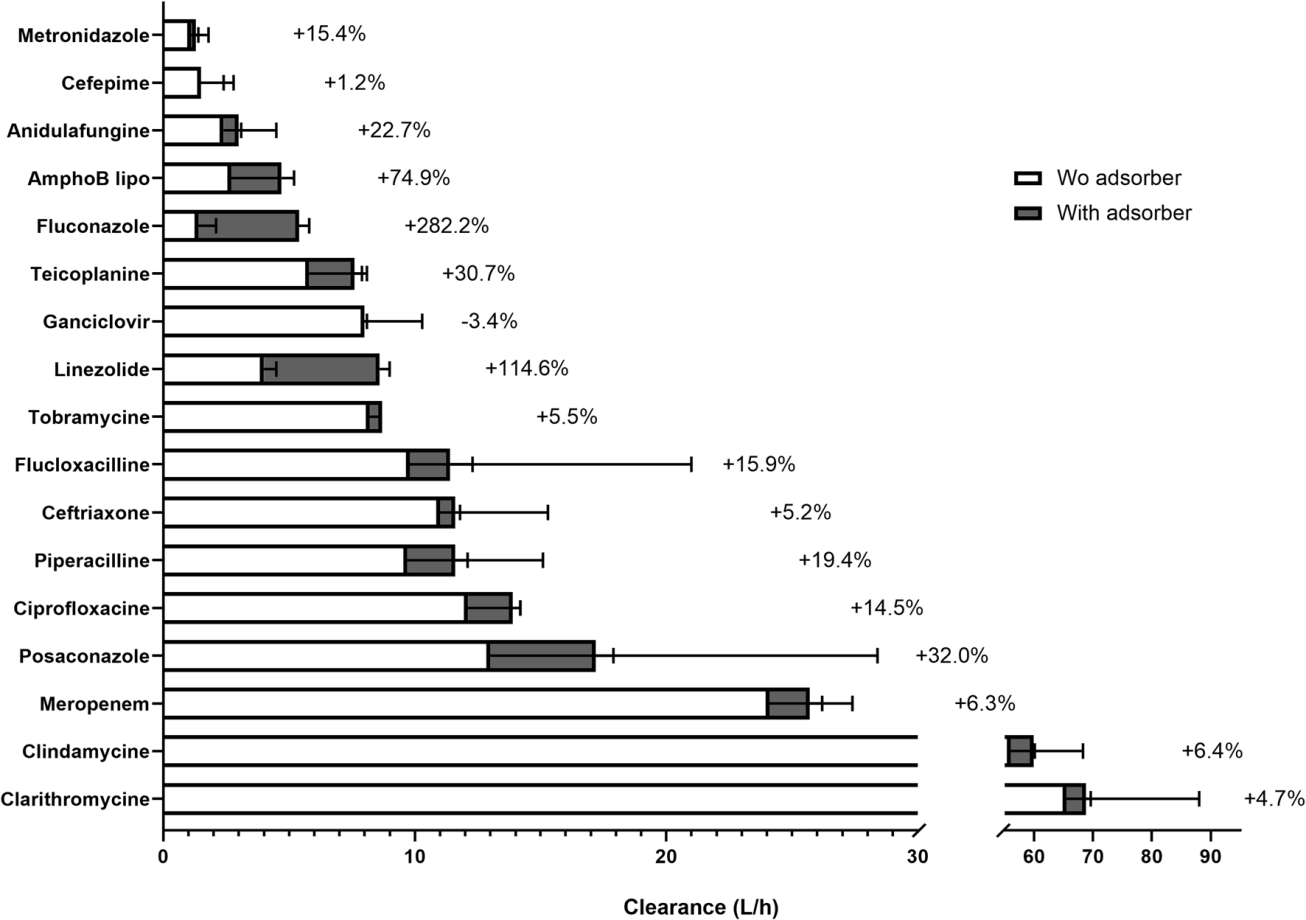
Additional clearance provided by adsorber. The white areas of the bars represent endogenous drug clearance (without adsorber) while the grey areas represent the additional clearance provided by the adsorber under the experimental conditions. Percentage refer to the relative increase in clearance associated with adsorber insertion. AmphoB lipo: liposomal amphotericin B.

Figure 3 presents for each study drug the impact of the hemoadsorber's associated clearance on total clearance. The hemoadsorber was associated with an increase in total clearance for all tested drugs (range 1 to 282%) except for ganciclovir (-3%). This increase was considered as moderate for fluconazole (282%) and linezolid (115%), mild for liposomal amphotericin B (75%), posaconazole (32%) and teicoplanin (31%) and negligible for all other drugs.

### Kinetics of adsorber associated clearance

**Figure 4:**
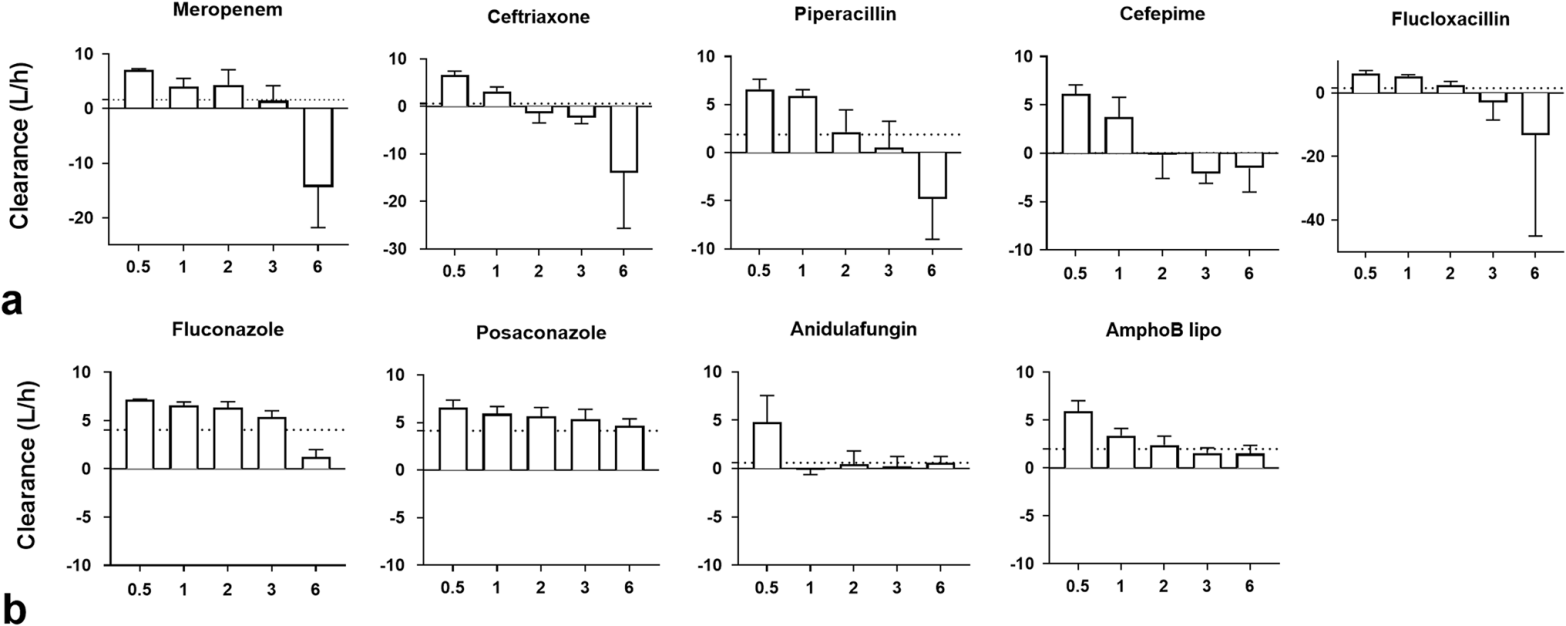
Kinetics of adsorber clearance of beta-lactams (**a**) and antifungals (**b**). Bars represent instantaneous clearance at the different study time points: 30 min (0.5 h), 1, 2 and 3 h after therapy initiation as well as the last measure obtained (6 h). Dotted lines represent total clearance (also represented in Fig. 2). Reported values are mean and standard deviation. AmphoB lipo: liposomal amphotericin B.

**Figure 5:**
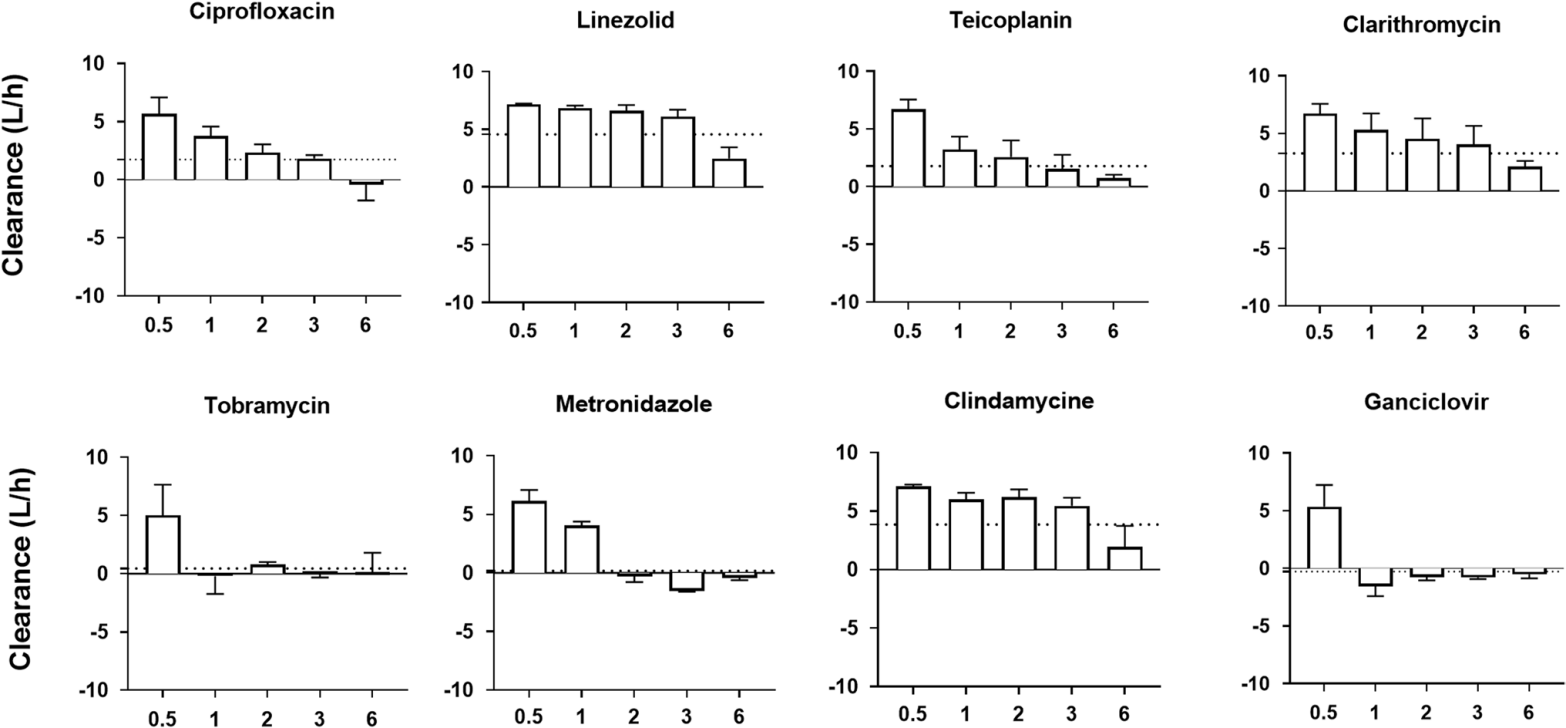
Kinetics of adsorber clearance of other anti-infective agents. Bars represent instantaneous clearance at the different study time points: 30 min (0.5 h), 1, 2 and 3 h after therapy initiation as well as the last measure obtained (6 h). Dotted lines represent total clearance (also represented in Fig. 2). Reported values are mean and standard deviation.

As depicted in Figs. 4 and 5, anti-infective clearance by the adsorber was not constant throughout the study period. For beta-lactams (piperacillin, flucloxacillin, ceftriaxone, cefepime and meropenem), it decreased progressively, even reaching negative values at the end of the experiment. For most other drugs (fluconazole, posaconazole, liposomal amphotericine B, linezolid, clarithromycin and teicoplanin), adsorber clearance decreased slowly but remained positive throughout the experiment. The clearance of some molecules became almost null respectively after one (anidulafungin, tobramycin, ganciclovir), two (metronidazole) or six (ciprofloxacin) hours of therapy and remained so for the rest of the experiment.

### Factors associated with overall hemoadsorber clearance

Overall hemoadsorber clearance appeared to increase proportionally with the octanol–water partition coefficient of drugs (logP) i.e. their lipophilicity (p= 0.01; r^2^=0.43), and to a lesser degree with the logD value (p=0.01; r^2^=0.36) (Fig. S5 of the supplementary material). There was no significant association with pKa, molecular electric charge, protein binding or molecular weight (all r^2^<0.1).

## Discussion

### Key findings

We have conducted an experimental study on 24 pigs to assess the impact of hemoadsorption with CytoSorb on the pharmacokinetics of a panel of anti-infective drugs commonly utilized in the management of patients with sepsis. We have found that for most drugs the procedure was associated with relatively high clearance. However, when endogenous clearance was considered, we found that the additional clearance provided was pharmacologically significant for only a few drugs (fluconazole, linezolid, liposomal amphotericin, posaconazole and teicoplanin) but negligible for other tested drugs. Importantly, we observed that the kinetics of clearance were not stable over time, with the highest clearance observed during the first hours of therapy, often followed by a rapid decline and even desorption for some drugs (in particular beta-lactams). Finally, we found that lipophilicity was the only pharmacokinetic factor moderately associated with overall hemoadsorber clearance.

### Comparison with previous studies

These first systematic data from in vivo experiments have to be compared to in vitro studies. Koenig et al. observed strong adsorption of anti-infectives to the CytoSorb adsorber with normal saline or human albumin as the perfusion fluid. Adsorption was markedly reduced for two anti-infectives, namely meropenem and ciprofloxacin, if reconstituted blood was used for perfusion. However, Koenig et al. integrated the adsorber into continuous veno-venous hemodialysis, so the results do not resemble pure hemoadsorption alone^14^. In addition, such data does not consider endogenous clearance and therefore the real impact of CytoSorb on in vivo pharmacokinetics is difficult to infer.

Other available data consist of case reports. The low clindamycin clearance observed in a young patient with refractory septic shock caused by Panton Valentin leucocidin producing methicillin resistant *Staphylococcus aureus* is consistent with the minimal effect of CytoSorb on clindamycin’s overall clearance (+4.7%) observed in our study^16^. Similarly, immediate removal of teicoplanin with a saturable process was described by Dimski et al^17^. This is in full agreement with our data, which shows high initial removal followed by progressive decline. Other observations have reported substantially lower linezolid peak levels during CytoSorb adsorption^15,21^. This is consistent with our finding of doubled clearance of this molecule during therapy.

Our finding of a paradoxical reduction of the systemic clearance of ganciclovir by the adsorber, corresponding to an apparent “creation” of drug in the cartridge, is in line with a release of drug from red blood cells, already observed during hemodiafiltration^22^.

A high affinity of CytoSorb for lipophilic molecules particularly in the range up to approximately 55 kDa has also been suspected^14,23^. We found a weak but significant correlation between the logP and hemoadsorber associated clearance. However, this property alone cannot be used to predict the impact of the hemoadsorber on drugs' pharmacokinetics. Indeed, when total clearance is considered, CytoSorb^'^ s effect on total clearance was limited for some drugs with high logP values (i.e. posaconazole, clarithromycin, flucloxacillin and clindamycin) while it was relatively important for some drugs with low logP values (fluconazole, linezolid or liposomal amphotericine B). This further highlights the need to consider hemoadsorber clearance relative to *overall* clearance.

Finally, in vitro studies have described CytoSorb 's adsorptive capacities as saturable^17^ and even subject to desorption (i.e. release of an adsorbed drug)^24^. Our study confirms these findings for the first time in vivo. The ability of polyvinylpyrrolidone, the substance covering CytoSorb's beads, to adsorb and desorb molecules has in principle been described in technical settings other than hemoadsorption, and has even been proposed for use as a component of pharmaceutical drug delivery systems^25^.

### Strengths and limitations

To the best of our knowledge, this is the first in vivo experimental study conducted to evaluate the impact of CytoSorb on the pharmacokinetics of anti-infective drugs. We tested a wide panel of medications commonly used in clinical practice. We have conducted sound analyses with thorough pharmacokinetics models. However, it has several limitations worth discussing.

First, our experimental study was conducted in healthy animals and our results might not directly be translatable to humans with septic shock. Indeed, drugs pharmacokinetics might vary from species to species. For instance, the clearances of ceftriaxone and teicoplanin measured in our model were higher than values previously reported in humans (respectively 8.4 vs 1.0 L/h and 5.4 vs 0.7-1.0 L/h) while that of linezolid was lower (8.8 vs 3.7 L/h)^26,27^. In addition, and more importantly, pharmacokinetic parameters are known to be massively altered in sepsis (increased volume of distribution, decreased protein binding etc.) particularly in cases of associated acute kidney injury or liver failure. In addition, in patients with sepsis, CytoSorb’s adsorptive capacities might be modified, typically by competitive adsorption of other molecules such as pro-inflammatory mediators. The net effect of such competition is unknown and might lead to desorption or decreased drug adsorption. Our model was designed to evaluate the isolated impact of hemoadsorption and minimize such confounders as much as possible. Further studies in humans with sepsis are therefore required to confirm or refute our findings.

Second, our protocol, enabled us to compute clearances obtained during the first 6 hours of CytoSorb adsorption, a duration lower than the manufacturer's recommended therapy (24 h). Hence, a delayed effect of CytoSorb therapy for instance total desorption (i.e. beta-lactams) or more significant binding (i.e. posaconazole) cannot be ruled out. However, our kinetics analyses strongly suggest that the majority of the adsorption process takes place in the first hours of therapy.

Third, medications were not administered separately but as a group, and their pharmacokinetics might have influenced each other through binding competition. However, our findings for drugs classes (beta-lactams and azoles) were robust and consistent, even if these medications were administered in different group combinations.

Last, administered doses of anti-infectives were higher than those typically recommended in clinical practice. We aimed to maximize serum concentration, based on the assumption that removal by the adsorber might be concentration dependent. This choice is likely to have biased our results toward an *increased* clearance attributable to the adsorber.

### Implications for clinicians and policy makers

Based on our data, some tentative recommendations can be formulated. Obviously, human studies should be performed before these recommendations are implemented in clinical practice.

The net influence of hemoadsorption with CytoSorb on beta-lactams' pharmacokinetics appears to be minimal. In addition, the initial adsorption followed by desorption might even be beneficial in terms of pharmacodynamics. Indeed, the intervention might optimize antimicrobial exposure and the activity of such time dependents agents. Hence, no dose adaptation would theoretically be required for this class of medications.

On the other hand, the observed initial removal of tobramycin, a concentration dependent drug, might be associated with a decrease in its antibacterial clinical efficacy. Here, a dose increase, accounting for the additional clearance, would be recommended. Temporary interruption of the adsorption therapy prior to drug administration could represent an alternative solution. The latter would have the theoretical advantage of enabling a high peak level (efficacy) and rapid removal (decreased toxicity).

For other drugs included in this study, biologic activity is related to the area under curve (AUC) divided by the minimum inhibitory concentration. This parameter was significantly decreased by CytoSorb hemoadsorption for three study drugs: fluconazole, linezolid and liposomal amphotericin B. Hence, decreased anti-infective activity is likely and drug dosage adaptation appears advisable. However, any required dose modification is likely to be minor. For all other drugs, the impact of the therapy on their total clearance was small and no major impact on pharmacodynamics would be expected and no dose adaptation would be required at least in patients with normal renal and liver function.

In acute kidney injury, or liver failure, the impact of hemoadsorption on total clearance is likely to increase making drug adaptation necessary. In these situations, therapeutic drug monitoring is strongly advised.

## Conclusions

Hemoadsorption with CytoSorb appears to have limited effect on the pharmacokinetics of the majority of drugs tested. However, the clearance of fluconazole, linezolid and liposomal amphotericin B appears to be increased by the procedure. These observations need to be confirmed during clinical trials conducted in humans with septic shock, to devise appropriate recommendations for dosage adjustment. In the meantime, therapeutic drug monitoring remains advisable.

## Supplementary Information

Supplementary Information.

## Data Availability

The datasets used and/or analyzed during the current study are available from the corresponding author on reasonable request.

## Abbreviations

HA: Hemoadsorption
ECC: Extracorporeal circulation
logP: Octanol–water partition coefficient
LogD: Octanol–water distribution coefficient
