# Supplementary material for: Pharmacokinetics of anti-infective agents during CytoSorb hemoadsorption
Source: Sci Rep. 2021 May 18;11:10493. doi: 10.1038/s41598-021-89965-z (PMC8131695; doi:10.1038/s41598-021-89965-z)
Supplement: Supplementary file 1 — Supplementary Information. [file 41598_2021_89965_MOESM1_ESM.docx]

# Pharmacokinetics of Anti-infective agents during CytoSorb Hemoadsorption

**Supplemental Material**

### Authors:

Antoine G. Schneider, MD, PhD ^1-2 *^ [antoine.schneider@chuv.ch](mailto:antoine.schneider@chuv.ch)

Pascal André, PharmD,PhD^3 *^ [pascal.andre@chuv.ch](mailto:pascal.andre@chuv.ch)

Joerg Scheier, MD ^4^ [joerg.scheier@cytosorbents.com](mailto:joerg.scheier@cytosorbents.com)

Monika Schmidt^5^ [m.schmidt@medizin-im-gruenen.de](mailto:m.schmidt@medizin-im-gruenen.de)

Heiko Ziervogel, PhD^5^ [h.ziervogel@medizin-im-gruenen.de](mailto:h.ziervogel@medizin-im-gruenen.de)

Thierry Buclin, MD^3^ [thierry.buclin@chuv.ch](mailto:thierry.buclin@chuv.ch)

Detlef Kindgen-Milles, MD ^6^ [kindgen-milles@med.uni-duesseldorf.de](mailto:kindgen-milles@med.uni-duesseldorf.de)

*: Both authors contributed equally to the work

1. Adult Intensive Care Unit, Centre Hospitalier Universitaire Vaudois (CHUV), Lausanne, Switzerland
2. Faculty of Biology and Medicine, University of Lausanne, Lausanne, Switzerland
3. Clinical Pharmacology, Centre Hospitalier Universitaire Vaudois (CHUV), Lausanne, Switzerland
4. CytoSorbents Europe GmbH, Berlin, Germany
5. Medical Competence Center Berlin/Brandenburg c/o HCx Consulting GmbH, Wendisch Rietz, Germany
6. Dept. of Anesthesiology, University Hospital Düsseldorf, Germany


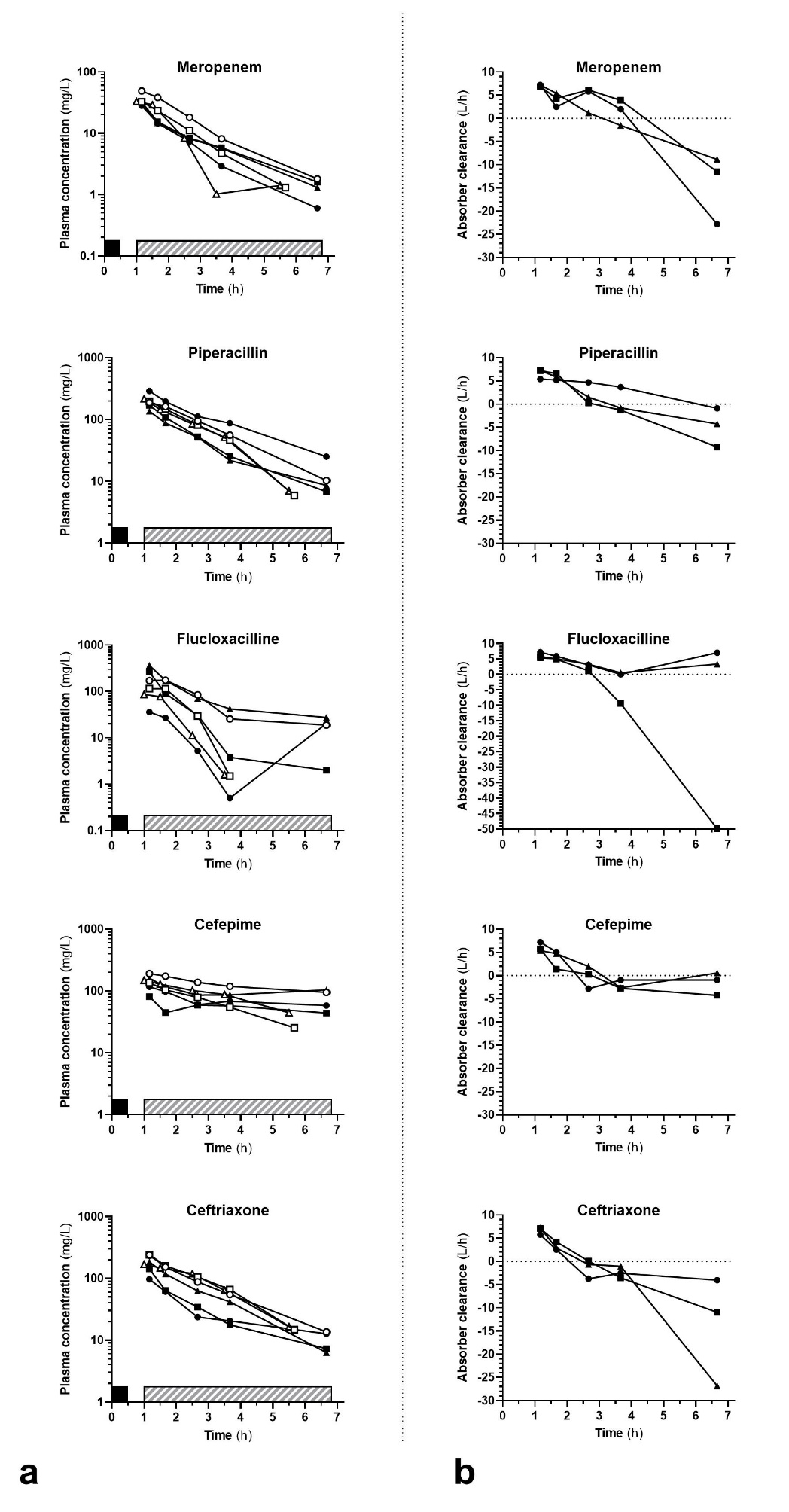


**Figure S1: Plasma concentrations (panel A) and calculated clearances (panel B) for beta-lactams.** Full symbols correspond to cases (CytoSorb Hemoadsorption) and open symbols to controls. Black box corresponds to drug administration and striped box to CytoSorb Therapy.


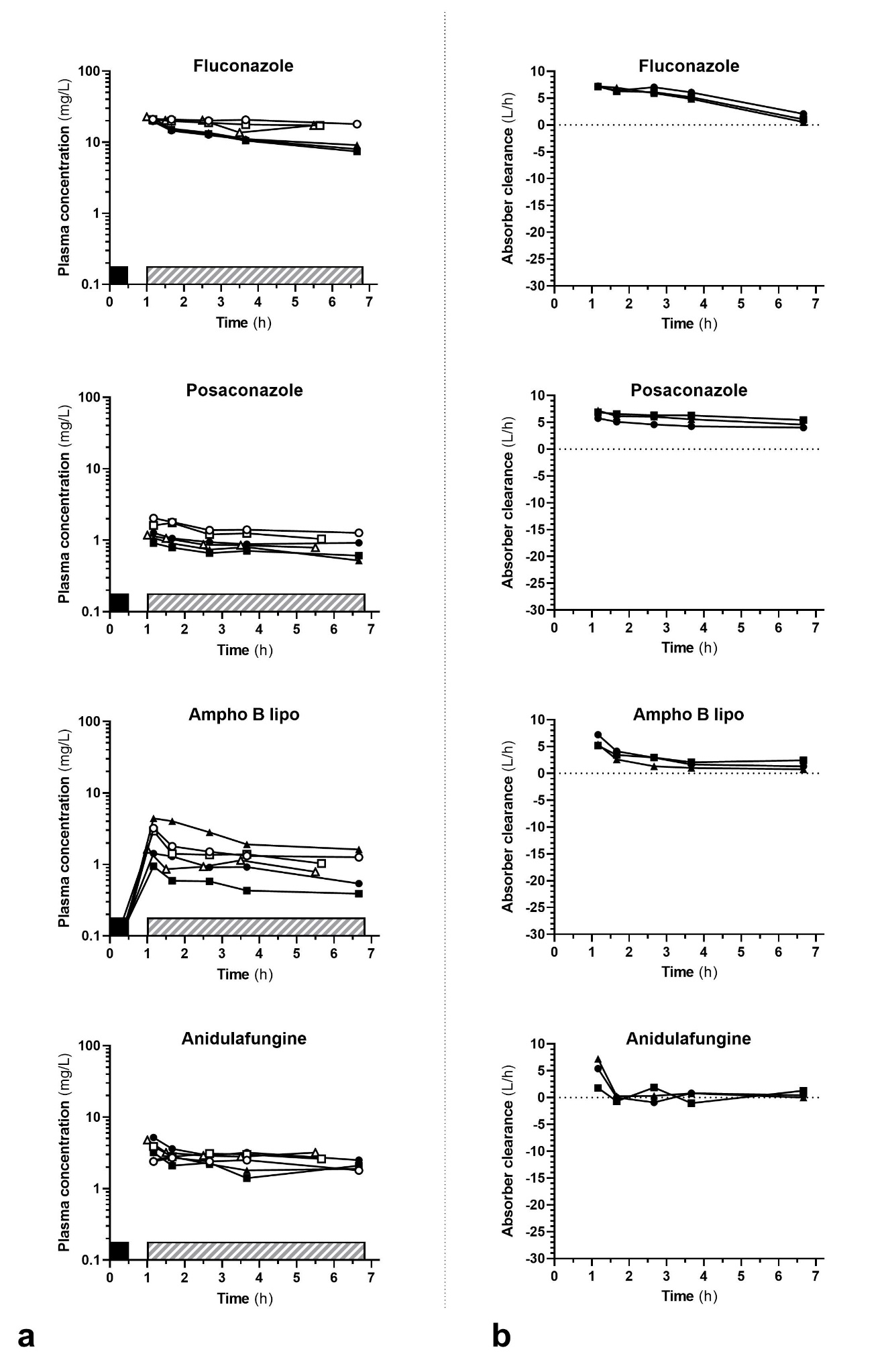


**Figure S2: Plasma concentrations (panel A) and calculated clearances (panel B) for antifungals.** Full symbols correspond to cases (CytoSorb Hemoadsorption) and open symbols to controls. Black box corresponds to drug administration and striped box to CytoSorb Therapy.


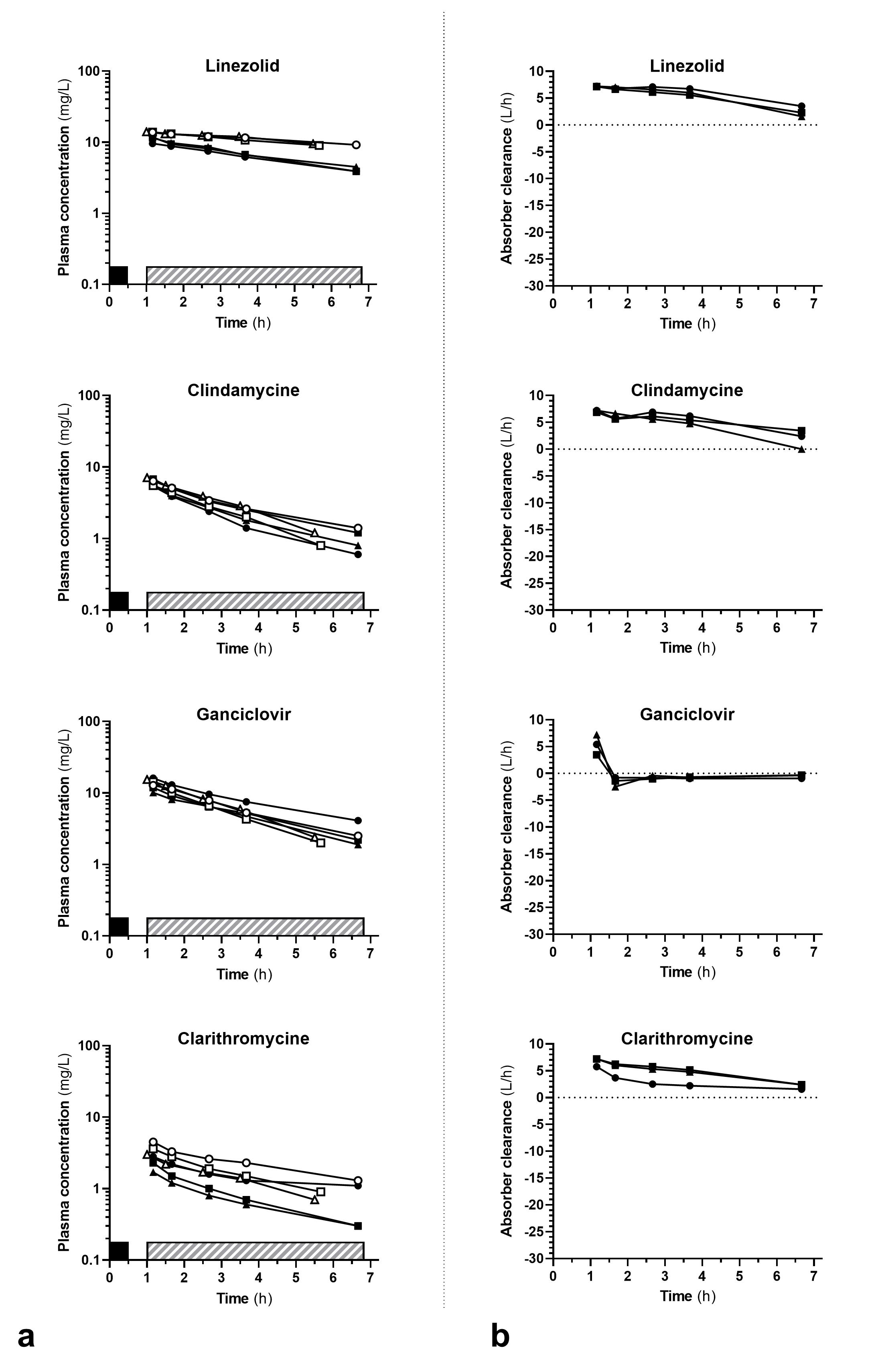


**Figure S3: Plasma concentrations (panel A) and calculated clearances (panel B) for other drugs.** Full symbols correspond to cases (CytoSorb Hemoadsorption) and open symbols to controls. Black box corresponds to drug administration and striped box to CytoSorb Therapy.


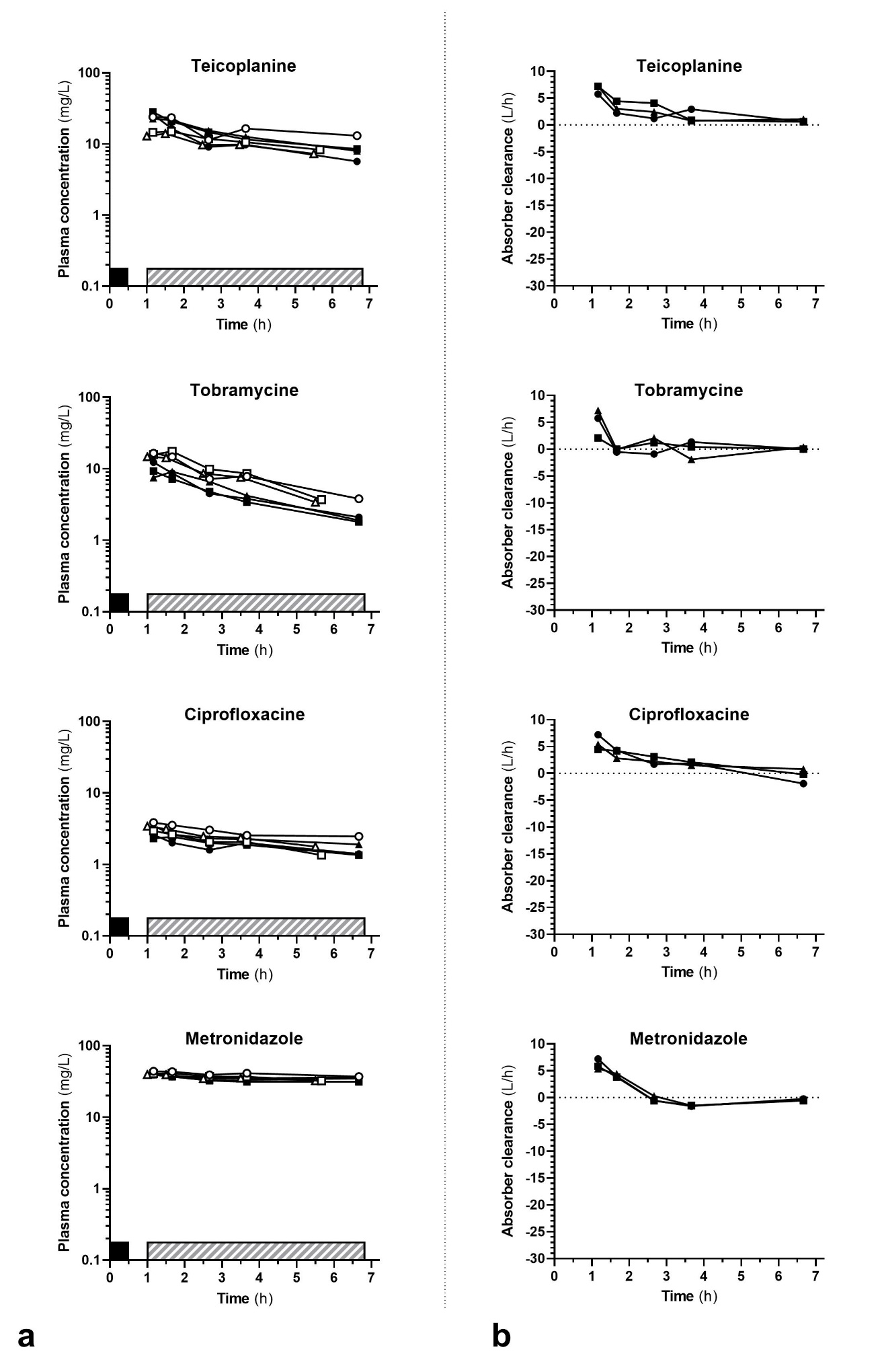


**Figure S4: Plasma concentrations (panel A) and calculated clearances (panel B) for other drugs.** Full symbols correspond to cases (CytoSorb Hemoadsorption) and open symbols to controls. Black box corresponds to drug administration and striped box to CytoSorb Therapy.


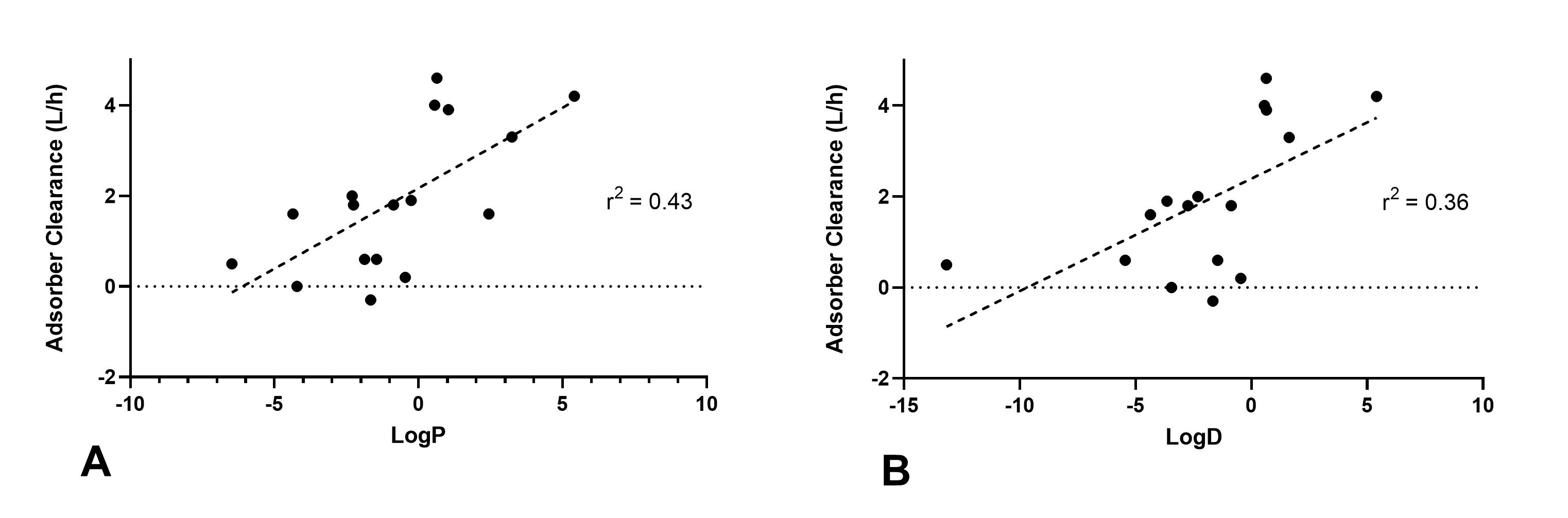


**Figure S5: Correlation plots.**

Correlation between drugs' partition coefficient (log P, panel A), distribution coefficient (logD, panel B) and absorber clearance. Correlations analyses performed using Pearson test, both p values < 0.05.
